# The impact of parental intimate partner violence and abuse (IPVA) on young adult relationships: a UK general population cohort study

**DOI:** 10.1016/j.lanepe.2025.101278

**Published:** 2025-05-01

**Authors:** Annie Herbert, Christine Barter, Eszter Szilassy, Jon Heron, Abigail Fraser, Maria Barnes, Alexa R. Yakubovich, Gene Feder, Laura D. Howe

**Affiliations:** a UK Medical Research Council Integrative Epidemiology Unit, University of Bristol, Bristol, UK; b Population Health Sciences, University of Bristol, Bristol, UK; c Centre for Academic Mental Health, University of Bristol, Bristol, UK; d Connect Centre, University of Central Lancashire, Preston, UK; e Centre for Academic Primary Care, University of Bristol, Bristol, UK; f Department of Community Health and Epidemiology, Dalhousie University, Halifax, Nova Scotia, United States; g MAP Centre for Urban Health Solutions, Unity Health Toronto, Toronto, Ontario, Canada

**Keywords:** Intimate partner violence and abuse, Intergenerational transmission, Measurement, Cohort studies, ALSPAC

## Abstract

**Background:**

There is uncertainty about the extent to which parental intimate partner violence and abuse (IPVA) increases risk of IPVA in the next generation. We aimed to provide estimates for the relationship between IPVA among mothers, and IPVA in their children’s own relationships as young adults.

**Methods:**

Using data from 3243 families from a UK birth cohort, we estimated risks of IPVA victimisation and perpetration among women and men aged 18–21 (a validated measure captured at age 21), according to mother’s IPVA victimisation status by age 18 (overall and separately for physical and psychological subtypes; a non-validated measure: 2–13 questions asked at ages 2, 4, 5, 8, 9, 11, 12, 18).

**Findings:**

Unadjusted relative risks (RR) for associations between maternal IPVA victimisation and subtypes of young adult IPVA ranged 0.91 to 1.54. There was a positive association between maternal psychological IPVA and subsequent victimisation among their children as young adult women (RR 1.23, 95% confidence interval [CI]: 1.07 to 1.41), attenuating to the null after adjustment for prenatal maternal IPVA and socio-economic factors. The strongest adjusted association was between maternal physical IPVA and perpetration among men (RR 1.45, 95% CI: 1.05 to 2.00). Maternal physical IPVA accounted for 10% of perpetration cases among men (CI: 2% to 16%). Most of this 10% was represented by young adults from families who experienced both maternal IPVA victimisation and child maltreatment.

**Interpretation:**

Interventions supporting young boys exposed to maternal physical IPVA could reduce risks of them using violence or abuse in their relationships. Services supporting families experiencing IPVA should consider co-occurring wider family adversity, which carried higher risk for intergenerational continuity of IPVA.

**Funding:**

10.13039/501100000265UK Medical Research Council (MR/S002634/1).

**Research in context**

**Evidence before this study**

We searched MEDLINE/PubMed for systematic reviews on the relationship between intimate partner violence and abuse (IPVA) across generations, published up to October 31, 2023, in English or French, given authors’ language abilities. Using terms related to “intimate partner”, “domestic”, “violence”, “abuse”, “victim”, “perpetration”, and “generation”, “transmission”, “family”, “risk factor”, and “outcome”, we identified reviews of risk factors for young adult IPVA, including parental IPVA, or outcomes of parental IPVA, including young adult IPVA. Inclusion criteria required primary studies from high-income countries, focusing on IPVA occurring between parents post-childbirth and before age 18, and IPVA in young adult relationships post-age 18. Narrative reviews without systematic searches, and opinion pieces, were excluded. We identified six relevant reviews. One review on parental IPVA’s impact on young adult IPVA perpetration found positive associations in 16 of 19 studies, with estimates ranging from a relative risk of 2.6 to an odds ratio (OR) of 4.4, and a strong focus on physical parental IPVA and North American populations. However, most studies had low methodological quality, were cross-sectional, and were based on retrospective reporting of parental IPVA. In contrast, two cohort studies found no evidence for an association. Another review conducted meta-analyses, reporting mean Cohen’s ds of 0.24 (95% Confidence Interval 0.20 to 0.27) for the relationship between witnessing interparental IPVA and young adult perpetration and 0.21 (0.18 to 0.24) for victimisation. The remaining four reviews included 2 to 4 studies on intergenerational effects not covered in previous reviews. These studies reported null to small positive associations (e.g. ORs up to 1.29), except one cross-sectional study suggesting a six-fold increased risk. The few longitudinal studies adjusted for family’s socio-economic background and child’s exposure to violence. The role of other adverse childhood experiences (ACEs) was unclear.

**Added value of this study**

This is the first prospective longitudinal study on intergenerational effects of IPVA in the UK. There were null and small positive associations for parent-child IPVA after adjusting for socio-economic circumstances and other ACEs. We report a small positive association between physical parental IPVA and IPVA perpetration among young men, consistent with the above literature. These associations persisted after adjusting for parental IPVA before the child was born, supporting social learning theory as an explanation. Our finding that the strongest association was between parental physical IPVA and IPVA perpetration among young men suggests that interventions targeting young boys could reduce involvement in IPVA later in life. Findings indicating that parental IPVA, combined with other forms of ACEs (predominantly child maltreatment), carried the highest risk, should guide how services supporting IPVA cases consider wider family adversity, and vice versa. Despite suggestions in the ACEs literature that parental IPVA, combined with substance misuse and mental health problems, is particularly harmful for children, our study found no evidence that this combination carried greater risks for IPVA. While up to 10% of IPVA perpetration cases could be accounted for by combinations of parental IPVA and other ACEs, up to 90% of young adult IPVA cases could not. This suggests that IPVA prevention efforts should not focus solely on subgroups defined by one or two exposures.

**Implications of all the available evidence**

In the Global North, among young men who grew up around physical IPVA between their parents, there is a moderately increased risk of IPVA perpetration within their own relationships. Although these associations are small, given large numbers of IPVA cases among parents, public health implications are substantial. These risks appear to be greater when children are exposed to both parental IPVA and maltreatment. Large longitudinal studies are needed to explore specific impacts of parental IPVA, such as frequency and severity, effects on different IPVA perpetration subtypes among men, and mediators like aggression or gender attitudes. Such research could provide further evidence supporting social learning theory for this intergenerational ‘transmission’. There is also a need for broader evidence on the complex network of other risk factors that may explain young adult IPVA, so that public health interventions can address the likely cumulative vulnerability that individual's experience.

## Introduction

Intimate partner violence and abuse (IPVA) between current or past intimate partners – affects 1 in 3 women globally.^1^^,^^2^ IPVA can also be referred to more simply as ‘intimate partner violence’ (‘IPV’); here the additional ‘abuse’ conveys that not all IPV is sexual or physical, the majority of cases will involve psychological abuse, including coercive or controlling behaviours.^3^^,^^4^ IPVA has the potential to be physically and psychologically damaging across generations.^2^^,^^5^^,^^6^ It is estimated that a quarter of UK adults have grown up in a household where IPVA has taken place.^7^

Childhood exposure to IPVA may place young people at greater risk of IPVA within their own relationships. Several systematic reviews of studies conducted during the past two decades, primarily focussing on cross-sectional studies in North America, conclude that children exposed to parental IPVA are at increased risk of IPVA in adulthood,^5^^,^^8^, ^9^, ^10^, ^11^, ^12^ with a dose–response relationship (increased risks with increased severity and frequency of parental IPVA),^5^ and with ‘social learning theory’ being a commonly hypothesised mechanism. According to social learning theory parents model behaviours for their children,^13^ and violence is normalised as a way of dealing with difficulty and conflict – this learning may be gendered, due to other childhood factors such as patriarchal cultures.^14^

To date, reported effect sizes for intergenerational effects of IPVA have varied from null to strong positive effects.^5^^,^^8^, ^9^, ^10^, ^11^, ^12^ An important issue likely influencing estimates, and highlighted by a methodological critique of these studies,^15^ is that parental IPVA is often captured via retrospective reports from the child when they reach adulthood, with likely differential under-reporting according to own IPVA status, and estimates distorted through recall bias. Further, there are inconsistencies regarding measurement of IPVA, including which time-points to consider, which are likely to account for some of the variation in estimates between studies.^15^ Whilst debates continue about the best way to measure IPVA, mixed-methods work, reviews, and international reports, generally agree that research needs to expand the focus beyond physical IPVA.^1^^,^^15^, ^16^, ^17^ In particular, they conclude that it should include controlling behaviours and impact as their consideration is rare,^15^, ^16^, ^17^ and that only ‘acts-based’ measures should be used as it captures more instances of IPVA than non-acts-based measures.^1^

Beyond accurately estimating the association between parental IPVA and later IPVA risks in the children, there is also a need for a better understanding of whether such a relationship is causal, which has rarely been a focus of these studies.^15^ A further limitation of the existing evidence is that it rarely takes account of other adverse childhood experiences (ACEs), which frequently co-occur with parental IPVA.^18^^,^^19^ Thus, it is not possible to evaluate the degree to which evidence for an increased risk of IPVA following parental IPVA is explained by a range of co-occurring family adversities. Elucidating whether parental IPVA increases the risk of IPVA in the next generation as they grow up, and the role of other co-occurring adversity, can tell us whether and how interventions or services supporting families or individuals who have grown up in abusive households, could reduce violence in the next generation.

Here, we studied the intergenerational effects of prospectively-reported IPVA in a UK general population birth cohort. We examined effects according to different subtypes of maternal IPVA victimisation (psychological [and to an extent, controlling behaviours], and physical) – which was captured using a mix of non-acts-based measure and acts-based measures – and young adult IPVA victimisation and perpetration (psychological [including controlling behaviours], physical, and sexual) – which were captured using a validated acts-based measure and with known underlying impact. We report findings by young adult’s sex (assigned at birth), adjusting for a range of factors during the mother’s pregnancy, including prenatal IPVA, and considering co-occurring family adversity.

## Methods

We analysed data on mother–child pairs from the Avon Longitudinal Study of Parents and Children (ALSPAC) cohort.^20^, ^21^, ^22^ Our study sample consisted of 3243 mother–child pairs where the child answered IPVA questions at age 21 (in 2012–2013), and neither had withdrawn consent by 2023.

### Original ALSPAC study data collection

All pregnant women residing in Avon, UK, with expected delivery dates in April 1991–December 1992 were eligible to participate in the original ALSPAC study, with around 14,500 women recruited (approximately three-quarters of the eligible population). The initial number of pregnancies enrolled was 14,541 (14,203 unique mothers), of which 13,988 children who were alive at 1 year of age. When the oldest children were approximately 7 years of age, an attempt was made to bolster the initial sample with eligible cases who had failed to join the study originally, resulting in an additional 913 children being enrolled. The total sample size for analyses using any data collected after the age of seven is therefore 15,447 pregnancies, of which 14,901 children were alive at 1 year of age. Information has been regularly collected since enrolment until the present. Given withdrawals of consent, data were available on 14,835 pregnancies (Table 1). The mothers and the children of the recruited pregnancies have been followed at least annually, through a mix of questionnaires and in-person clinics.

**Table 1:** Characteristics of families in the current study sample (n = 3243), compared to those recruited at baseline in ALSPAC (n = 14,835).

	Women				Men			
	Study sample (n = 2104)		Baseline ALSPAC (n = 7258)		Study sample (n = 1139)		Baseline ALSPAC (n = 7577)	
**Parents (age of child/young adult when measured)**								
Mother’s marital status in pregnancy								
Married	1752	83.4	5417	74.6	981	86.4	5594	73.8
Sexual orientation (by age 7)								
Not 100% heterosexual	29	1.3	78	1.1	19	1.7	76	1.0
Smoked in pregnancy								
Yes	479	22.8	2435	33.6	227	20.0	2654	35.0
Age of mother at delivery in years (birth)	29	26 to 32	28	25 to 31	30	27 to 33	28	25 to 31
Parity (birth)								
0	997	47.4	3307	45.6	590	52.0	3377	44.6
1	750	35.7	2511	34.6	362	31.9	2630	34.7
2	263	12.5	1010	13.9	142	12.5	1091	14.4
3+	92	4.4	430	5.9	42	3.7	479	6.3
Mother’s highest education level (birth)								
≥A-level	918	47.5	2117	35.7	575	53.8	2198	34.9
Household highest social class (birth)								
I (most affluent)	373	17.8	876	12.1	266	23.4	960	12.7
II	960	45.7	2931	40.4	528	46.5	3008	39.7
III non-manual	502	23.9	1892	26.1	251	22.1	1975	26.1
III manual	193	9.2	1051	14.5	66	5.9	1128	14.9
IV or V (most deprived)	73	3.5	507	7.0	24	2.1	505	6.7
IPVA (0–18 y)								
Any	998	47.5	3599	49.6	509	44.9	3740	49.4
Physical	517	24.6	2040	28.1	269	23.7	2074	27.4
Psychological (any)	827	39.4	3026	41.7	422	37.2	3144	41.5
Controlling behaviours	162	7.7	528	7.3	82	7.2	603	8
**Child/young adult (age when measured)**								
Ethnicity (birth)								
Person of Colour	1992	95.8	6422	94.2	1082	96.3	6782	94.3
Sexual orientation (21 y)^a^								
Not 100% heterosexual	354	16.8	.		157	13.8	.	
Weight (g, birth)	3400	3081 to 3688	3376	3041 to 3678	3522	3200 to 3860	3480	3121 to 3820
IPVA (18–21 y)^a^								
Any victimisation	674	32.1	.		270	23.8	.	
Physical	266	12.7	.		86	7.6	.	
Psychological (any)	564	26.8	.		243	21.4	.	
Coercive control	359	17.1	.		155	13.7	.	
Sexual	247	11.8	.		45	4.0	.	
Any perpetration	437	20.8	.		177	15.6	.	
Physical	150	7.1	.		20	1.8	.	
Psychological (any)	401	19.1	.		165	14.5	.	
Controlling behaviours	208	9.9	.		80	7.0	.	
Sexual	<5	0.2	.		15	1.3	.	
Adverse Childhood Experiences (ACEs; 0–16 y)								
Maltreatment	949	45.2	3182	43.8	511	45	3303	43.6
Emotional abuse	420	20.0	1387	19.1	220	19.4	1425	18.8
Emotional neglect	372	17.7	1437	19.8	240	21.1	1733	22.9
Physical abuse	406	19.3	1184	16.3	216	19.1	1040	13.7
Sexual abuse	106	5.0	240	3.3	15	1.4	73	1.0
Bullying	433	20.6	1511	20.8	302	26.6	1982	26.2
Parental mental health problems	861	41.0	3228	44.5	449	39.6	3216	42.4
Parental substance misuse	169	8.0	651	9.0	88	7.8	735	9.7
Parental criminal conviction	128	6.1	530	7.3	73	6.4	520	6.9
Parental divorce/separation	526	25.0	2119	29.2	254	22.4	2064	27.2
Combinations of ACEs (0–16 y)^b^								
4 or more ACEs (any)	230	11.0	874	12.0	127	11.2	932	12.3
4 or more ACEs (one being Parental IPVA)	186	8.9	685	9.4	97	8.5	718	9.5
4 or more ACEs (two being Parental IPVA + Maltreatment)	183	8.7	644	8.9	94	8.3	669	8.8
4 or more ACEs (three being Parental IPVA + Parental mental health problems + Parental substance misuse)	44	2.1	163	2.2	24	2.1	174	2.3
Parental IPVA + Maltreatment	549	26.1	1840	25.3	277	24.4	1906	25.2
Parental IPVA + Parental mental health problems + Parental substance misuse	67	3.2	249	3.4	32	2.8	256	3.4

ACEs = Adverse Childhood Experiences; IPVA = Intimate Partner Violence & Abuse; y = years.

Descriptive statistics are pooled estimates across 35 imputed datasets.

a Sexual orientation and young adult IPVA reported at age 21 only, therefore numbers for the ‘Baseline ALSPAC’ columns would be the same, but percentages would artificially appear a lot smaller.

b ‘+’ meaning that they co-occurred together.

### Current study sample

The sample of 3243 mother–child pairs represents 21% of the original 15,447 pregnancies, and 35% of the 9359 contactable at age 21. Based on questionnaire meta-data, we estimate that for around 5% of the study sample, a non-biological mother responded to at least one of the questionnaires at which parental IPVA was captured – we include all these main caregivers on the basis that the child will learn behaviours from their environment that they will carry into adulthood, regardless of biological parenthood. Amongst the pregnancies, there were 37 sets of twins. One ‘child’ (young adult) was randomly selected from each set for inclusion in the mother-child pair. From our previous work in this sample, using measures that likely under-capture intimate relationships, we can confirm that at least 88% of these young adults have had an intimate encounter.^23^

### Terminology

Here, time-points represent approximate age of the child/young adult (intended age at that wave, most surveys occurred within one year of this intended age). We refer to main caregivers of these young people (whether the biological mother or not) as the *mother* and main caregivers and their partner(s) (whether biological father or not), as the *parents*.

Supplementary Fig. S1 shows a Directed Acyclic Graph of hypothesised relationships between key exposure, outcome, and confounder variables.

### Exposure: maternal (partner-to-mother) IPVA victimisation

The exposure was any IPVA victimisation of the mother by their partner, a binary yes/no measure representing any victimisation by the time the child was age 18, based on information reported by the mother in surveys when the child was aged 2, 4, 5, 8, 9, 11, 12, and 18 years. We note that these questions and combining them into one binary measure of any IPVA by age 18, have not been validated.

Question and response wording, and how responses were treated in analyses, are provided in Supplementary Table S1. Briefly, when the child was aged 2, 4, 5, 9, 11, and 18, the mother was asked if their ‘husband/partner’ had been ‘cruel’ to them, emotionally or physically (respectively), since the previous survey time-point (ranging from past eight months to three years). At child ages 8 and 12, the mother was asked about experiencing specific behaviours: at age 8 they were asked about 13 different behaviours from their husband/partner relating to psychological or physical IPVA (e.g. ‘Has your husband/partner insulted or shamed you in front of others?’ [psychological]; ‘Have you ever been pushed, grabbed or shoved by your husband/partner?’ [physical]); at age 12, they were asked about experiencing two physical IPVA behaviours in the past three months. At age 12, they were also asked about four controlling behaviours in the past three months (‘my husband/partner … ’ ‘ … wants to know exactly what I’m doing and where I am’; ‘insists I do exactly as I’m told’; ‘seeks to dominate me’; ‘tends to control everything I do’).

When studying maternal IPVA by subtype, we analysed three separate binary variables, representing any psychological IPVA across the seven relevant time-points at which it was captured, any physical IPVA across eight time-points, and any controlling behaviours at one time-point (age 12). Controlling behaviours were considered a sub-category of psychological IPVA, and any reporting of controlling behaviours was included in psychological IPVA’s measurement.

### Outcome: young adult IPVA

IPVA victimisation and perpetration were reported by the ALSPAC children at age 21. In contrast to the maternal IPVA exposure (which captured psychological and physical IPVA victimisation each with one crude non-validated question at six of the eight time-points), the young adult IPVA outcome was captured in response to twelve questions about four, two, and two examples of psychological, physical, and sexual IPVA victimisation, and one, one, and two examples of psychological, physical, and sexual IPVA perpetration, respectively (exact questions and response wording in Supplementary Box S2). The questions were developed based on UK and European questionnaires and have been previously validated.^24^^,^^25^ The questions also asked whether these examples occurred before or after turning 18, or at both time-points.

Victimisation and perpetration were analysed as separate binary outcomes, defined as any IPVA occurring after 18 (i.e., including responses of occurring both before and after 18). Thresholds were a response of at least ‘once’, to any of the eight victimisation questions, and to any of the four perpetration questions, respectively. The rationale was that the questionnaire section header was ‘Intimate Partner Violence’, likely raising the threshold of severity for reporting certain behaviours, and that 75–99% of participants answering ‘at least once’ also reported negative impact.^23^

### Covariates

We included the following potential confounders^11^^,^^12^ of the maternal IPVA-young adult IPVA relationship in adjusted analyses (all recorded during pregnancy or at time of birth and largely indicators of socio-economic background): highest of parents’ education, highest of parents’ social class; mother’s: marital status, smoking status, depression score, age at delivery, parity; birthweight; and prenatal maternal IPVA (reported at 18 weeks gestation). Ethnicity was collected in ALSPAC and considered a potential confounder. However, 95% of the young adults were recorded as being ‘White’ and ethnicity was omitted from final analyses, as models did not converge when the variable was included. Beyond the covariates mentioned above, unplanned pregnancy is considered a strong risk factor for IPVA among women,^11^ however this was not available among the ALSPAC mothers prenatally. After adjusting for other covariates, we do not perceive any ‘back door path’ between unplanned pregnancy and young adult IPVA (Supplementary Fig. S1).

To inform whether co-occurrence of ACEs affects the parental-young adult IPVA association, models were further adjusted for nine binary indicators of other ACEs occurring at age 0–16: adult-to-child emotional neglect, emotional abuse, physical abuse, or sexual abuse; bullying from peers; parental mental health problems, parental substance abuse, parental criminal conviction, or parental separation. These ACEs were developed previously, capturing information from 374 variables across different time-points in the ALSPAC data.^7^ For these variables the term ‘parental’ is used to mean mother, biological father, or mother’s partner.

More details on how the above covariates were measured and treated in analyses are in Supplementary Table S2.

### Statistical analyses

We calculated descriptive statistics of all variables for the analysis sample and, where possible, the full ALSPAC cohort. We examined the association between maternal IPVA (overall and by maternal IPVA subtype) and young adult IPVA by estimating risk-ratios (RRs) from modified Poisson regression models; Adjusted Model A includes standard confounders as defined under *Covariates*; Adjusted Model B additionally includes ACEs. We used modified Poisson (also known as ‘robust Poisson’) models they are a suitable alternative to log-binomial models when estimating relative risks, with fewer issues of convergence, when the outcome is common (the prevalence of the outcome was up to 32%; Table 1).^26^^,^^27^ Given the complex bi-directional relationship between maternal IPVA and other ACEs, estimates from Adjusted Model A are intended to be interpreted as potentially causal; those from Adjusted Model B are not. We provide unadjusted estimates within results tables to allow comparison with estimates in existing literature. Given that the maternal IPVA victimisation exposure was captured via a mixture of non-acts-based measures (e.g. ‘Your partner was physically cruel to you’) and more objective acts-based measures (e.g. ‘Has your partner pushed, grabbed, or shoved you?’), we repeated the above analyses restricting the exposure to only acts-based measures (indicated in Supplementary Table S1).

Finally, we used adjusted RRs to calculate Population Attributable Fractions (PAFs), indicating the proportion of cases of young adult IPVA that can be accounted for by the occurrence of maternal IPVA. Details on how PAFs, accompanying risk differences (RDs), and confidence intervals (CIs) were calculated are in Supplementary Box S3. PAFs and RDs are presented for any maternal IPVA victimisation and subtypes at all, and then in combination with the following ACE categories: maltreatment (adult-child emotional neglect, emotional abuse, physical abuse, sexual abuse); three or more other ACEs (i.e., 4+ ACEs, when in combination with maternal IPVA); parental mental health problems + parental substance abuse.^19^^,^^28^ These ACE categories are commonly researched and suggested as targets for public health intervention; they also commonly co-occur with IPVA.^18^ Therefore, findings may be used to inform services supporting families experiencing IPVA and/or other ACEs as to who may be at particularly high risk of experiencing future violence and abuse.

Some models that included all confounders would not converge when estimating adjusted RRs for IPVA in combination with other adversities, given overlap between the definitions of some confounders and ACEs. Therefore, for estimating PAFs and RDs, we reduced the set of model covariates to a binary indicator of household social class (I-IIINM vs. IIIM-V), prenatal smoking status, maternal age, and prenatal maternal IPVA – based on capturing a broad range of socio-economic indicators (e.g. maternal age is known to highly correlate with parity and so only one of these variables were taken forward), and using the least sparse covariate measures available. Analyses were carried out in Stata 17 (scripts provided at www.github.com/pachucasunrise/IPVA_intergen). Given known differing IPVA patterns reported by women and men, and to allow comparison of findings with relevant literature studying women only,^5^^,^^11^ we stratified all analyses by sex assigned at birth (gender identity was not available from the data).

Missing data were imputed using multiple imputation with chained equations (MICE). Proportions of missing data prior to multiple imputation for exposures, outcomes, and covariates used in analyses are reported in Supplementary Table S3. In our study sample, before imputation, maternal IPVA was missing for 13% of the sample when the children were age 2, generally increasing with age to 37% at age 18 (possibly due to attrition of the original parents from the study over time). Given that maternal IPVA was captured across multiple questions over time from birth to age 18, we considered values for the binary variable of Maternal IPVA to be missing if less than 50% of the component questions had been answered (if ≥ 50% of questions answered, and: all questions answered had negative responses = 0, at least one of the questions had a positive response = 1). We used this 50% criteria when creating other related IPVA variables. For example, the binary variable of Psychological Maternal IPVA was considered non-missing if at least 50% of its 19 component questions (see Supplementary Table S1) had been answered. Given criteria for study inclusion, there were no missing data for young adult IPVA.

Following this, we carried out MICE to impute missing values of maternal IPVA exposure, young adult IPVA outcomes, and covariates. We assume that Maternal IPVA data are Missing At Random given information from covariates and auxiliary variables included in imputation models. We created 35 imputed datasets. Imputations were stratified by sex, and included all exposures, outcomes, and covariates of interest (including the 9 ACEs listed in Supplementary Table S2 and included in Adjusted Model B [see first paragraph within this ‘Statistical analyses' section]). We also included whether mother smoked in pregnancy or not and the child’s birthweight as auxiliary variables. Within the same models, we passively imputed the binary ACE categories of child maltreatment (any emotional abuse, emotional neglect, physical abuse, or sexual abuse), 3+ ACEs other than maternal IPVA, and Parental mental health problems or substance abuse. We pooled estimates (%s, RRs, PAFs, and RDs) across imputed datasets using Rubin’s rules.^29^ Results for complete cases are reported in Supplementary Table S4. There was no loss to follow-up given eligibility criteria that the young adult had answered IPVA questions by age 21.

### Role of the funding source

The funders (UK Medical Research Council) had no role in study design, data collection and analysis, decision to publish, or preparation of the manuscript.

## Results

The final analysis sample was 2104 women and 1139 men aged 18–21, and their mothers (Table 1). The approximate 2:1 ratio of women to men in the current study sample is a result of study attrition and non-response to the age 21 questionnaire, consequences of which are later discussed under *Strengths and limitations*.

Mothers gave birth to the ALSPAC children at a median age of 29–30 (IQR: 26–27 to 32–33; statistics are presented disaggregated by sex of the children), with half having already birthed an older sibling. They were relatively well educated and affluent compared to the general population or originally recruited mothers (around half had ≥A level qualifications; around two-thirds were in the highest two of five possible household classes). Half of the sample parents had suffered either mental health problems, substance misuse, or criminal conviction by the time the children were age 16; one quarter had separated (including divorce). For nearly half of the children, some form of maltreatment was reported by age 16, for nearly one-quarter, bullying. IPVA and other adverse experiences were slightly less likely in families within the final analysis sample compared to those that were originally recruited. Table 1 provides descriptive statistics about the study sample, alongside those for the baseline ALSPAC cohort.

### Descriptive statistics of parental and young adult IPVA

Of the ALSPAC mothers, 45–48% reported IPVA victimisation, the most common subtype being psychological (Table 1; including imputed values, psychological: 37–39%, physical: 24–25%, controlling behaviours: 7–8%). Around one-third of the ALSPAC children suffered IPVA victimisation and one-fifth reported perpetration in their young adult relationships. These rates were higher in young women compared with young men (victimisation: 32% vs. 24%, perpetration: 21% vs. 16%).

### Association of maternal IPVA with young adult IPVA victimisation, overall and by IPVA subtype

**Fig. 1:**
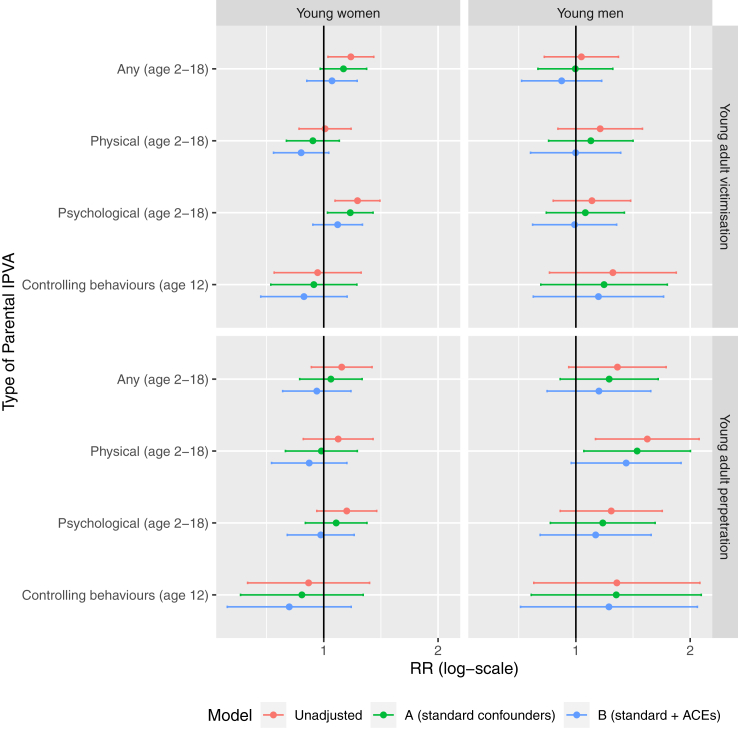
Associations of parental intimate partner violence and abuse (IPVA) with young adult IPVA victimisation, overall (any parental IPVA) and by IPVA type (e.g., psychological). ACEs: Adverse Childhood Experiences; IPVA: Intimate partner violence and abuse; RR: Relative Risk.

Young women growing up with mothers in a violent or abusive relationship were more likely to be victimised in their own intimate partner relationships as young adults (crude relative risk [RR] for any maternal IPVA: 1.18, 95% CI: 1.03 to 1.35; Fig. 1, Supplementary Table S5). For young men, risks were similar between those with exposed and unexposed mothers. However after adjustment for prenatal parent and child factors (i.e., our best estimates of causal associations), the association among women attenuated to the null (adjusted Model A RR: 1.13, 0.98 to 1.30).

When estimates for these associations were broken down by subtype, CIs were very wide. When the violence or abuse was psychological, point estimates were similar to those for overall IPVA, as expected given that psychological IPVA was the most prevalent subtype. Among women, there was little evidence of an association between maternal and young adult IPVA when the maternal IPVA was either physical or controlling behaviours in both unadjusted and adjusted models; among men, the RR was highest when the maternal IPVA was physical (unadjusted RR: 1.16, adjusted Model A: 1.10; Fig. 1, Supplementary Table S5), but the 95% CIs were wide and spanned the null. After adjustment for co-occurring ACEs estimates generally attenuated further towards the null (adjusted Model B). Findings were similar when analyses were carried out in complete cases (Supplementary Table S4). When the exposure was restricted to acts-based measures of maternal IPVA victimisation, findings were similar except for the association between psychological maternal IPVA victimisation and young adult IPVA victimisation among women, where estimates moved closer to the null (adjusted Model A: 0.99, 0.85 to 1.16; Supplementary Table S4).

### Association of maternal IPVA with young adult IPVA perpetration, overall and by IPVA subtype

There was little evidence for an increased risk of perpetrating IPVA in women exposed to maternal IPVA, compared to those unexposed (unadjusted RR: 1.12, 0.93 to 1.34, Fig. 1, Supplementary Table S6). Conversely, men who had been exposed were at an increased risk of perpetration (1.29, 0.96 to 1.73). The association between maternal IPVA and IPVA perpetration was stronger if the maternal IPVA was physical (1.54, 1.13 to 2.11). Effect estimates attenuated but remained positive after adjustment for socio-economic and prenatal factors (adjusted Model A: 1.45, 1.05 to 2.00) and for co-occurring ACEs (adjusted Model B: 1.36, 0.97 to 1.89), in the latter case the CI included unity. Associations with perpetration outcomes were similar in complete cases, and when using a tighter acts-based measure of maternal IPVA victimisation (Supplementary Table S4).

### Burden of maternal IPVA in the context of young adult IPVA (Population Attributable Fractions)

PAFs in Tables 2 and 3 indicate that maternal IPVA accounted for 6% of cases of IPVA victimisation among young women (1% among men) and 9% of perpetration cases among men (2% among women), after adjusting for other factors that may explain these relationships. However, CIs were wide and spanned the null. When PAFs were broken down by maternal IPVA subtypes, the highest was for physical IPVA, accounting for 10% of IPVA perpetration cases among young men (95% CI: 2%–16%) (Table 2).

**Table 2:** Population Attributable Fractions and Risk Differences of Maternal IPVA victimisation and other ACEs for IPVA victimisation in young adulthood.

Factor	Women						Men					
	Prevalence of exposure (%)	PAF (%)	(95% CI)	Risk in reference group (no IPVA; %)	Risk difference (% points)	(95% CI)	Prevalence of exposure (%)	PAF (%)	(95% CI)	Risk in reference group (no IPVA; %)	Risk difference (% points)	(95% CI)
**Maternal IPVA (age 2–18)**^a^	47.5	5.82	(−1.1 to 11.82)	30	5.3	(3.3–7.2)	44.9	<0.1	(−11.8 to 9.2)	23	0.8	(−0.8 to 2.4)
+ Maltreatment	26.1	5.1	(0.6–9.0)		6.0	(0.4–11.6)	24.4	3.1	(−4.8 to 9.1)		2.3	(−4.7 to 9.4)
+3 or more other ACEs^b^	16.0	4.1	(0.8–6.9)		8.1	(1.3–15.0)	15.2	3.5	(−2 to 7.6)		4.9	(−3.6 to 13.4)
+ MH + SA	3.2	0.4	(−1.5 to 1.5)		3.5	(−9.5 to 16.6)	2.8	<0.1	(−4.1 to 1.7)		−0.8	(−17.5 to 15.8)
**Maternal Physical IPVA (age 2–18)**^a^	24.6	<0.1	(−6.3 to 2.2)	31	0.3	(−2.4 to 2.9)	23.7	2.3	(−4.8 to 7.8)	23	3.7	(1.9–5.4)
+ Maltreatment	11.2	<0.1	(−3.1 to 2.1)		−0.8	(−7.9 to 6.4)	10.9	3.0	(−1.4 to 6.1)		5.4	(−4 to 14.9)
+ 3 or more other ACEs^b^	6.9	1.1	(−0.9 to 2.6)		5.4	(−3.7 to 14.4)	7.7	2.6	(−0.9 to 5.1)		7.0	(−4 to 18.0)
+ MH + SA	1.4	0.2	(−1.2 to 0.9)		3.2	(−17.3 to 23.6)	1.6	<0.1	(−3.6 to 1.2)		−1.1	(−22.2 to 20.1)
**Maternal Psychological IPVA (age 2–18)**^a^	39.4	6.6	(1.0–11.5)	29	6.7	(4.7–8.7)	37.2	2.2	(−7.8 to 10.1)	23	2.4	(0.7–4.0)
+ Maltreatment	17.3	4.5	(1.5–7.1)		8.2	(2.1–14.4)	17.6	4.4	(−0.9 to 8.5)		5.6	(−2.0 to 13.2)
+ 3 or more other ACEs^b^	11.1	3.7	(1.5–5.6)		11.0	(3.5–18.4)	10.9	3.6	(−0.3 to 6.3)		7.6	(−1.8 to 17.0)
+ MH + SA	2.5	0.7	(−0.7 to 1.6)		9.6	(−5 to 24.3)	2.0	<0.1	(−3.6 to 0.9)		−4.3	(−21.9 to 13.2)
**Controlling behaviours between parents (age 12)**^a^	7.7	<0.1	(−2.8 to 1.4)	32	−1.1	(−3.9 to 1.6)	7.2	1.4	(−2.1 to 3.8)	23	5.9	(4.2–7.7)
+ Maltreatment	3.2	0.1	(−1.3 to 1.1)		1.3	(−10.9 to 13.5)	3.5	2.0	(0.2–3.1)		12.8	(−3.0 to 28.7)
+ 3 or more other ACEs^b^	1.8	<0.1	(−1.2 to 0.7)		0.2	(−16.2 to 16.6)	2.3	1.7	(0.4–2.5)		17.1	(−2.9 to 37.1)
+ MH + SA	0.5	<0.1	(−2.0 to 0.3)		−8.2	(−37.4 to 21.0)	0.5	0.3	(−1 to 0.6)		15.4	(−27.7 to 58.5)

ACEs = Adverse Childhood Experiences; aRR = adjusted relative risk; CI = Confidence Interval; IPVA = Intimate Partner Violence & Abuse; LCI = Lower Confidence Limit; MH = Mental health problem; PAF = Population Attributable Fraction; SA = Substance abuse; UCI = Upper Confidence Limit.

PAFs calculated as p(1-1/aRR), where p is the prevalence of the factor of interest (e.g. emotional neglect) among those exposed to the outcome. Confidence intervals for PAFs calculated as p(1-1/LCI) and p(1-1/UCI). aRRs estimated from modified Poisson models and RDs from binary logistic regression models. Both sets of models adjusted for age of mother at delivery (in years), whether mother smoked in pregnancy or not, household social class (I; II; III non-manual; III manual; IV or V), and any maternal IPVA during pregnancy (yes/no). Estimates are pooled estimates across 35 imputed datasets.

a Any, regardless of whether other ACEs present.

b Including: emotional neglect, emotional abuse, physical abuse, sexual abuse, parental substance abuse, parental mental health problems, parental criminal conviction, parental separation, and bullying.

**Table 3:** Population Attributable Fractions of Maternal IPVA victimisation and other ACEs for IPVA perpetration in young adulthood.

Factor	Women						Men					
	Prevalence of exposure (%)	PAF (%)	(95% CI)	Risk in reference group (no IPVA; %)	Risk diff. (% points)	(95% CI)	Prevalence of exposure (%)	PAF (%)	(95% CI)	Risk in reference group (no IPVA)	Risk difference (% points)	(95% CI)
**Maternal IPVA (age 2–18)**^a^	47.5	2.1	(−7.9 to 10.4)	20	2.3	(0.6–4.0)	44.9	9.4	(−5.1 to 20.1)	14	4.0	(2.6–5.3)
+ Maltreatment	26.1	2.0	(−5.0 to 7.6)		1.6	(−3.2 to 6.4)	24.5	11.5	(2.3–18.0)		7.3	(1.2–13.4)
+3 or more other ACEs^b^	16.0	2.9	(−2.1 to 6.7)		3.9	(−2.0 to 9.8)	15.2	8.1	(1.3–12.6)		8.2	(0.8–15.6)
+ MH + SA	3.2	0.2	(−1.8 to 1.5)		1.7	(−10.0 to 13.4)	2.8	<0.1	(−12.4 to 0.8)		−5.9	(−16.4 to 4.5)
**Maternal Physical IPVA (age 2–18)**^a^	24.6	<0.1	(−6.9 to 4.8)	20	1.9	(−0.2 to 4.0)	23.7	10.0	(1.5–16.2)	14	7.5	(5.9–9.1)
+ Maltreatment	11.2	0.8	(−3.0 to 3.7)		1.2	(−5.1 to 7.6)	10.9	8.9	(4.4–11.9)		12.4	(3.6–21.2)
+3 or more other ACEs^b^	6.9	1.9	(−0.8 to 3.8)		5.7	(−2.6 to 14.0)	7.7	7.0	(3.4–9.4)		13.6	(3.5–23.8)
+ MH + SA	1.4	0.5	(−0.7 to 1.1)		6.5	(−12.9 to 25.9)	1.6	<0.1	(−10.4 to 0.6)		−4.9	(−18.4 to 8.6)
**Maternal Psychological IPVA (age 2–18)**^a^	39.3	3.1	(−5.0 to 9.8)	20	2.9	(1.2–4.7)	37.2	6.4	(−7.1 to 16.2)	14	3.4	(1.9–4.9)
+ Maltreatment	17.3	2.3	(−2.5 to 6.1)		2.8	(−2.5 to 8.0)	17.6	8.0	(1.0–12.8)		6.9	(0.3–13.5)
+ 3 or more other ACEs^b^	11.1	2.8	(−0.7 to 5.4)		5.5	(−1.0 to 12.0)	10.9	4.3	(−1.1 to 7.8)		6.1	(−1.8 to 13.9)
+ MH + SA	.	.	.		.	.	.	.	.		.	.
**Controlling behaviours between parents (age 12)**^a^	7.7	<0.1	(−4.7 to 1.5)	21	−1.8	(−3.9 to 0.3)	7.2	2.0	(−2.8 to 4.8)	15	4.4	(2.6–6.1)
+ Maltreatment	3.2	0.3	(−1.6 to 1.4)		2.4	(−8.4 to 13.1)	3.5	2.5	(0.2–3.8)		11.7	(−2.4 to 25.8)
+ 3 or more other ACEs^b^	1.8	0.4	(−1.0 to 1.2)		5.3	(−10.6 to 21.1)	2.3	1.6	(−0.3 to 2.6)		12.2	(−4.9 to 29.3)
+ MH + SA	.	.	.		.	.	.	.	.		.	.

ACEs = Adverse Childhood Experiences; aRR = adjusted relative risk; CI = Confidence Interval; IPVA = Intimate Partner Violence & Abuse; LCI = Lower Confidence Limit; MH = Mental health problems; PAF = Population Attributable Fraction; RD = Risk Difference; UCI = Upper Confidence Limit; . = could not be estimated, model did not converge.

PAFs calculated as p(1-1/aRR), where p is the prevalence of the factor of interest (e.g. emotional neglect) among those exposed to the outcome. Confidence intervals for PAFs calculated as p(1-1/LCI) and p(1-1/UCI). aRRs estimated from modified Poisson models and RDs from binary logistic regression models. Both sets of models adjusted for age of mother at delivery (in years), whether mother smoked in pregnancy or not, household social class (I; II; III non-manual; III manual; IV or V), and any maternal IPVA during pregnancy (yes/no). Estimates are pooled estimates across 35 imputed datasets.

a Any, regardless of whether other ACEs present.

b Including: emotional neglect, emotional abuse, physical abuse, sexual abuse, parental substance abuse, parental mental health problems, parental criminal conviction, parental separation, and bullying.

Of the young adults included in our analysis, 26% grew up around both maternal IPVA and child maltreatment (Table 2). However, when using this co-occurrence as the exposure rather than any maternal IPVA, the PAFs for the outcome of IPVA in young adulthood were largely unaltered (e.g. 3–5% for IPVA victimisation among both men and women vs. up to 6%). Similar patterns were observed for individuals experiencing both maternal IPVA and 3 or more other ACEs (this combination had a prevalence of 16%). For 91% of the children experiencing maternal IPVA in combination with 3 or more other ACEs, maltreatment was one of these other ACEs (Table 1). PAFs of young adult victimisation and perpetration, for maternal IPVA combined with parental mental health problems and substance abuse, were relatively low (<0.1%–0.4%) with low prevalence (3%) (Tables 2 and 3).

## Discussion

In this study, the objective was to estimate the intergenerational effects of prospectively-reported IPVA, according to maternal IPVA subtypes and young adult sex. We found a modest association between maternal IPVA (partner-to-mother victimisation) and IPVA victimisation among their children as young adult women, and between maternal IPVA and IPVA perpetration in young adult men. After adjustment for potential confounding factors, the association for victimisation among women attenuated towards the null, but a weak positive association for perpetration among young men remained (RR: 1.22), consistent with some previous reviews of studies on intergenerational effects of IPVA.^8^^,^^11^ The strongest association was for perpetration by men growing up around physical IPVA victimisation (RR: 1.45). Another objective was to consider co-occurring family adversity - up to 10% of young adult IPVA cases were accounted for by prior maternal IPVA, with a large majority of these cases also experiencing childhood maltreatment. Findings can inform preventative interventions focussing on young boys growing up around IPVA, particularly in the context of broader child maltreatment.

The study addresses an important limitation of previous research: retrospective reporting in adulthood of childhood exposure to IPVA, likely leading to distorted estimates of an intergenerational effect.^15^^,^^30^ In our cohort, parental IPVA was prospectively-reported by the mother, and reported regularly from the child’s birth to adulthood, rather than being retrospectively reported by their children as young adults. The measurement of maternal IPVA was not validated and had limitations. Most questions were rudimentary (i.e., captured by a single question at each time-point and with questions open to interpretation, e.g. ‘has your partner ever been emotionally cruel to you?’), and were more open to interpretation than the young adult IPVA questions – nevertheless there was still an association with perpetration among young men when the maternal IPVA victimisation exposure was restricted to a subset of more objective (though still unvalidated) acts-based measures. The parental IPVA questions did not capture crucial information such as severity, frequency, or impact.^31^^,^^32^ Therefore, there was potential for misclassification of maternal IPVA, with victimisation being under-reported through for example, social desirability bias or not perceiving IPVA behaviours as violent or abusive.^16^^,^^25^ Potential mis-classification is further compounded by gender: women are less likely than men to perceive their own experiences as victimisation and more likely to perceive their behaviour as perpetration.^16^^,^^25^ Since parental IPVA was mother-reported and all questions related to victimisation (Supplementary Table S2), these issues will further contribute to under-capture of parental IPVA. In contrast, questions on IPVA among the young adults were carefully developed and validated,^25^ and recall bias (about IPVA in the past three years) is likely to be minimal. Although the occurrence of maternal IPVA was captured at several time-points, data were not available on whether the child witnessed this IPVA. According to Holden’s taxonomy,^33^ there are several ways that a child can be ‘exposed’ to parental IPVA postnatally and at different levels: for example, intervening themselves, not seeing the incident but overhearing, or hearing about the assault from another family member. It is possible that some maternal IPVA cases were within families where children were ‘ostensibly unaware’ – such cases would render associations in this study underestimates of the relationship between witnessing parental IPVA and young adult IPVA.

A key strength was the rich data available to facilitate investigating: causality (through temporal separation of exposure and outcome); consistency with social learning theory (by isolating exposure to postnatal maternal IPVA and adjusting for prenatal maternal IPVA); different maternal IPVA subtypes; and co-occurrence of a range of prospectively-reported ACEs. However, there were limitations to maternal IPVA subtype data, and other key covariates. Controlling behaviours, reported once, were likely under-reported, with a study prevalence of 7%. Parental sexual IPVA was not reported at all by age 21. Coercive control and sexual IPVA victimisation are subtypes predominantly experienced by women,^34^ and so this would likely result in further under-estimation of intergenerational effects for this group. It was not possible to stratify analyses by gender identity or sexual orientation of either parents or their children, where minority groups are estimated to have higher prevalence of IPVA.^35^ Gender identity of either parents or young adults were not enquired about by the time the young adult was age 21. We used sex assigned at birth as a proxy for young adult gender. Whilst sexual orientation of both parents and young adults were indicated by age 21 (the parents at time of child’s birth and when age 7; the young adult at age 15 and 21), the numbers of those reporting minority status were too small to be able to stratify and produce stable estimates (Table 1).

Most ‘intergenerational transmission’ research has recruited clinical or student populations^15^; our study is from a general population cohort, and does include participants who did not attend university. ALSPAC has been shown to originally well represent the Avon area from which it was recruited at the time; though conversely this means it has higher proportions of people from high socio-economic position than the UK population.^21^ There were also high rates of attrition – only 21% of the original pregnancies are represented by the young adults who responded at age 21, and compared to the baseline cohort had higher levels of socio-economic indicators. There was a gender imbalance in this attrition where men were more likely to be lost to the study: at baseline in ALSPAC the children were 49% female; by age 21 this increased to 65%. Overall rates of parental and young adult IPVA in the study sample, and patterns of IPVA subtypes, corresponded with other UK general population estimates.^24^ We would expect the most extreme cases of parental and young adult IPVA to be those lost to the study, resulting in underestimated associations, and potentially more so among young men.

In summary, given under-capture of maternal IPVA victimisation (particularly coercive control, and non-capture of sexual IPVA), and gendered misclassification of IPVA, it is likely that the associations between maternal IPVA and subsequent IPVA victimisation among women, and perpetration among men, are under-estimated. However, given the breadth of IPVA data and covariates included, these estimates get us closer to a general population estimate than was previously available.^17^ It is likely that exposure to physical parental IPVA truly increases risks of IPVA perpetration among young men, given the strength of association, combined with existing feminist and social learning theories regarding gender norms and dealing with conflict.^13^

Findings of small associations for victimisation among young women and perpetration among young men,^8^ are consistent with systematic reviews of previous studies.^5^^,^^8^, ^9^, ^10^, ^11^, ^12^ To date, only a handful of studies have explored intergenerational effects whilst identifying a theoretical framework, some of which have included social learning as the proposed mechanism.^10^ The current study extends that work, which was all cross-sectional, and only included physical maternal IPVA exposures and IPVA perpetration outcomes. By including adjustment for prenatal IPVA, our study findings are consistent with social learning theory.

The literature has been less clear on risks associated with maternal IPVA in combination with other ACEs, which our study found to be higher when that combination included child maltreatment. Despite prior suggestions in the ACEs literature that parental IPVA in combination with parental substance misuse and parental mental health problems is particularly harmful for children,^28^ there was no evidence of greater risks of IPVA for these children later in life.

The association between maternal IPVA and IPVA in young adult relationships was largely explained by adjustment for confounders including socio-economic indicators and prenatal IPVA. This implies that addressing maternal IPVA is unlikely to be effective for primary prevention of young adult victimisation or young women’s perpetration. The association of maternal IPVA with men’s IPVA perpetration remained, meaning that interventions that focus on boys exposed to parental IPVA, such as improved education around sex, relationships, and masculinity,^36^ or aggression and conflict,^37^ may be effective in breaking the cycle of perpetration.^38^ Identifying potential mediators on the parent-young adult IPVA pathway among men, such as their understanding and attitudes on gender or ways of dealing with conflict,^39^ will be important to support the design of such interventions.

PAFs for young adult IPVA were similar for maternal IPVA overall (i.e., regardless of other exposures) and for maternal IPVA in combination with 3 or more other ACEs. That is, intergenerational effects of IPVA were largely accounted for by a subset of high-risk families, where the children had also been exposed to other ACEs (predominantly child maltreatment). This emphasises the importance of services that support families affected by IPVA considering wider trauma. It further emphasises the vulnerability of children from families with complex adversity to unhealthy relationships later in life. It should also be noted, however, that up to 90% of young adult IPVA cases could not be explained by combinations of IPVA, other ACEs, and a range of early life factors – therefore, IPVA prevention efforts cannot be directed based on a single exposure only, and measures that tackle a range of pathways to IPVA are needed. This includes broader systemic and contextual factors (e.g., poverty, access to affordable and comprehensive services) that underpins family violence.

Future research is needed to address two areas this study could not. First, this study was limited in its ability to study intergenerational effects of maternal sexual IPVA or coercive control. This is important given that children are common targets of coercive control alongside and independently of their parent.^40^ Second, larger sample sizes, realistically only possible through multi-country collaborations, can help to provide sufficiently precise estimates and therefore definitive conclusions regarding: parental IPVA exposures that not only considers subtype but other important factors such as severity and impact; different young adult IPVA outcomes (including IPVA subtypes that may give further insights regarding social learning theory); and mediators informing about potential intervention targets. Given the imperfect measurement of maternal IPVA in the present study, which will have contributed to measurement error and variability of estimates, data that better captures IPVA in multiple generations will both reduce bias and improve precision of estimates. This would be possible through identifying new data sources or improving measurement of IPVA in ongoing data collections.^17^ Qualitative work can enhance understanding in all these areas, particularly how parental IPVA and other adversity such as maltreatment impacts the child’s experiences of relationships (romantic and otherwise) leading into adulthood.^41^ Finally, there is a need for broader evidence on the likely complex network of other risk factors and mediators that may explain young adult IPVA, to shed light on other possible mechanisms, such as compromised emotional development,^42^ or general strain theory (i.e., increased emotional strain on the individual [due to the parental IPVA itself but also through its wider impacts on the individual’s experiences and goals] leading to increased likelihood of perpetration as a means of coping).^30^^,^^43^ Better understanding of this network can help public health interventions ‘chip away’ at the likely cumulative vulnerability that individuals experience.

In summary, using prospectively-reported maternal IPVA victimisation measures and validated young adult IPVA measures, we observed small positive associations between maternal IPVA and IPVA in young adult relationships for psychological victimisation among women, and for perpetration following maternal physical IPVA among men. These increased risks are small in magnitude but likely to contribute towards large numbers of young adult IPVA cases given high prevalence of maternal IPVA. We showed that maternal IPVA victimisation is responsible for up to 10% of cases of IPVA in young adult relationships, largely concentrated amongst young people who experienced complex adversity including child maltreatment. Therefore, services supporting domestic violence cases in families should consider wider trauma, including history of child maltreatment.

## Contributors

CB, JH, AF, ES, GF, and LDH conceptualised this study and acquired funding. AH, JH, AF, and LDH had access to all the data. AH conducted all data analyses and wrote the original draft. All authors interpreted study findings, contributed to reviewing and editing the work, and approved the final version. AH agrees to be accountable for all aspects and had final responsibility for the decision to submit for publication.

## Data sharing statement

Information on how to access Avon Longitudinal Study of Parents & Children (ALSPAC) data is available in Supplementary Box S1. If you have any further questions about accessing data, please email alspac-data@bristol.ac.uk.

## Declaration of interests

This work was funded by the UK Medical Research Council (MRC; Grant reference: MR/S002634/1, PI GF, Co-Is: ES, AF, LDH, JH, CB). ES receives grant funding from UK National Institute for Health & Care Research (NIHR), and received funding from National Health Service Bristol, North Somerset and South Gloucestershire Integrated Care Board at the time of the study. AF receives grant funding from British Heart Foundation (BHF), UK Research & Innovation, Leducq Foundation, and Templeton Foundation, and sits on UK MRC and Wellbeing of Women boards. GF receives grant funding from UK Prevention Research Partnership and NIHR Public Health Research Programme, and is non-executive board member of the social enterprise, IRIS-i. LDH receives grant funding from Wellcome, BHF, UK Economic and Social Research Council, and UK MRC. None of the above funding bodies or grant panels had no role in study design, data collection and analysis, decision to publish, or preparation of the manuscript.

## Appendix ASupplementary data

Supplementary Fig. S1, Boxes S1–S3, and Tables S1–S6
