# Supplementary material for: The impact of parental intimate partner violence and abuse (IPVA) on young adult relationships: a UK general population cohort study
Source: Lancet Reg Health Eur. 2025 May 1;53:101278. doi: 10.1016/j.lanepe.2025.101278 (PMC12237738; doi:10.1016/j.lanepe.2025.101278)
Supplement: Supplementary Fig. S1, Boxes S1–S3, and Tables S1–S6 [file mmc1.docx]

**Supplementary materials**

- Box S1: Further details on the ALSPAC study and data access
- Figure S1: Hypothesised Directed Acyclic Graph (DAG) of causal relationships between variables of interest.
- Table S1: Variables capturing mother-reported parental IPVA in ALSPAC.
- Box S2: Intimate Partner Violence and Abuse (IPVA) section of questionnaire delivered to ALSPAC ‘children’ (as young adults) at age 21.
- Table S2: Variables used to capture covariates.
- Box S3: Details on deriving Population Attributable Fractions (PAFs), Risk Differences (RDs) and their Confidence Intervals (CIs).
- Table S3: Proportions of missing data in exposures, outcomes, and covariates, prior to multiple imputation
- Table S4: Association of parental IPVA* with young adult IPVA**, overall (any parental IPVA) and by IPVA type (e.g. psychological), in complete case (n=953 to 1878) vs. imputed data (n, women=2104, men=1139; including when exposure restricted***)
- Table S5: Associations of parental IPVA with young adult IPVA victimisation, overall (any parental IPVA) and by IPVA type (e.g. psychological)
- Table S6: Associations of parental IPVA with young adult IPVA perpetration, overall (any parental IPVA) and by IPVA type (e.g. psychological)
- References

**Box S1: Further details on the ALSPAC, derivation of study sample, and data access**

**Data collection and management**

Ethical approval for the study was obtained from the ALSPAC Ethics and Law Committee and the Local Research Ethics Committees. Informed consent for the use of data collected via questionnaires and clinics was obtained from participants following the recommendations of the ALSPAC Ethics and Law Committee at the time.

Study data were collected and managed using REDCap electronic data capture tools hosted at the University of Bristol.(1) REDCap (Research Electronic Data Capture) is a secure, web-based software platform designed to support data capture for research studies. The study website contains details of all the data that are available through a fully searchable data dictionary and variable search tool (<http://www.bristol.ac.uk/alspac/researchers/our-data/>).

**Data access**

ALSPAC data access is through a system of managed open access. The steps below highlight how to apply for access to ALSPAC data, including access to the Stata/R scripts used for analyses reported in this Research Article.

1. Please read the [ALSPAC access policy (PDF, 627kB)](http://www.bristol.ac.uk/media-library/sites/alspac/documents/researchers/data-access/ALSPAC_Access_Policy.pdf) which describes the process of accessing the data and samples in detail, and outlines the costs associated with doing so.

2. You may also find it useful to browse our fully searchable [research proposals database](https://proposals.epi.bristol.ac.uk/), which lists all research projects that have been approved since April 2011.

3. Please [submit your research proposal](https://proposals.epi.bristol.ac.uk/) for consideration by the ALSPAC Executive Committee. You will receive a response within 10 working days to advise you whether your proposal has been approved.

If you have any questions about accessing data, please email [alspac-data@bristol.ac.uk](mailto:alspac-data@bristol.ac.uk).

The ALSPAC data management plan describes in detail the policy regarding data sharing, which is through a system of managed open access.


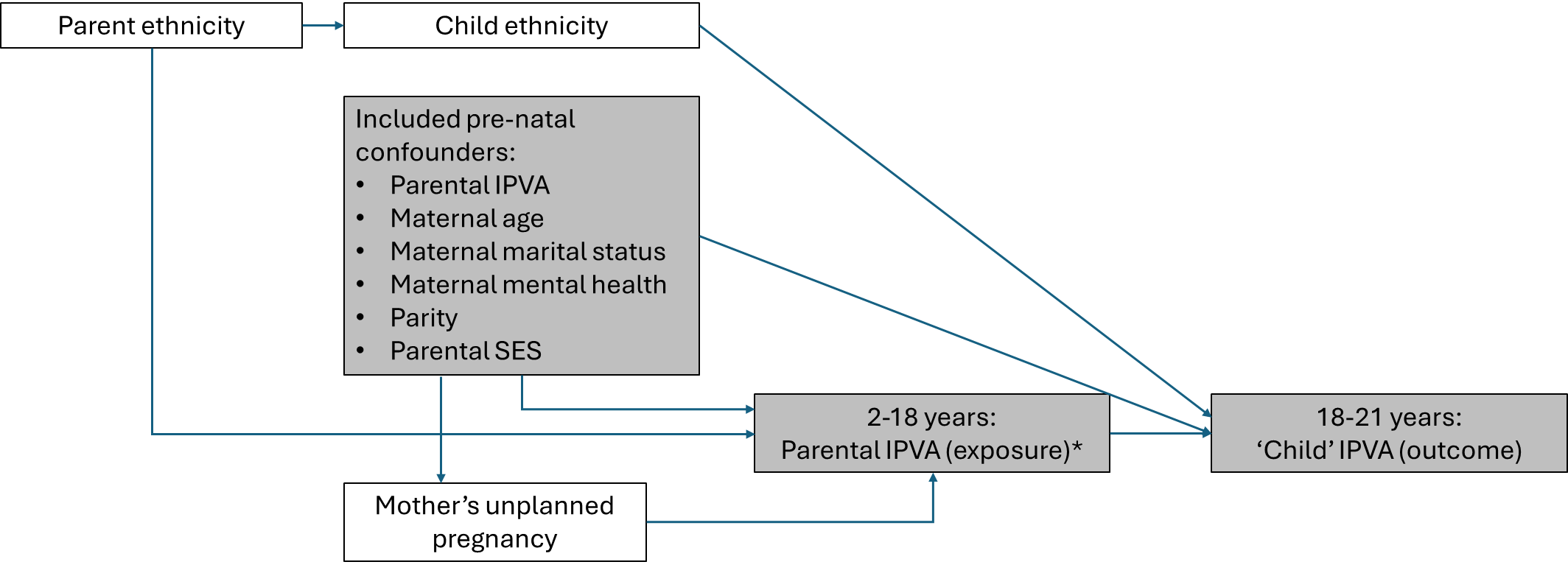


IPVA: Intimate partner violence and abuse; SES: Socio-economic status

*Adverse Childhood Experiences at age 0-16 were also included in analyses – it has a complex bidirectional relationship with Parental IPVA and so we have not depicted here on the DAG.

N.B. Time-points refer to birth/age of G1 (the ‘children’ of the ALSPAC birth cohort). Filled boxes include variables that were included in analyses. Though pre-natal parental IPVA is not available in ALSPAC data, we assume that it has a path to young adult IPVA (the outcome), only through either parental IPVA during pregnancy (included in adjustments) or post-natal parental IPVA (the exposure), and thus that assume we are not omitting a relevant confounder. Hypothesised relationships between ethnicity and IPVA, based on Field *et al* and Cho *et al;*(2, 3) hypothesised relationships between pre-natal confounders and IPVA based on Capaldi *et al* and Yakubovich *et al*.(4, 5)

**Figure S1: Hypothesised Directed Acyclic Graph (DAG) of causal relationships between variables of interest**

**Table S1: Variables capturing mother-reported parental IPVA in ALSPAC**

| **ALSPAC variable** | **Age of ALSPAC child** | **Question** | **Parental IPVA sub-type** | **How used in current study** | **Response options, split into a binary outcome (where used in study)** |
| --- | --- | --- | --- | --- | --- |
| b592 | 18w gestation | "Have any of these occurred since you became pregnant? If so, please assess how much effect it had on you." …"Your partner hurt you" | Physical | Covariate | 1 = Y affected a lot; Y moderately affected; Y mildly affected; Y but did not affect me;  0 = N did not happen at all |
| b607 | 18w gestation | "Have any of these occurred since you became pregnant? If so, please assess how much effect it had on you." …"Your partner was emotionally cruel to you" | Psychological | Covariate |  |
| h232a | 2.75y | “Listed below are a number of events which may have brought changes in your life. Have any of these occurred since the baby was 18 months old? If so, please assess how much effect it had on you.” …”Your partner was physically cruel to you.” | Physical | Exposure | 1 = Y affected a lot; Y moderately affected; Y mildly affected; Y but did not affect me;  0 = N did not happen at all |
| h246a | 2.75y | Listed below are a number of events which may have brought changes in your life. Have any of these occurred since the baby was 18 months old? If so, please assess how much effect it had on you. …”Your partner was emotionally cruel to you.” | Psychological | Exposure |  |
| j322a | 4y | “Listed below are a number of events which may have brought changes in your life. Have any of these occurred since the baby was 2 ½ years old? If so, please assess how much effect it had on you.” …”Your partner was physically cruel to you.” | Physical | Exposure |  |
| j336a | 4y | Listed below are a number of events which may have brought changes in your life. Have any of these occurred since the baby was 2 ½ years old? If so, please assess how much effect it had on you. …”Your partner was emotionally cruel to you.” | Psychological | Exposure |  |
| k4022 | 5y | “Have any of the these occurred in the past year (since your study child was 4). Some of these may be distressing to recall, but we hope you will let us know just how they affected you.” …”Your partner was physically cruel to you” | Physical | Exposure |  |
| k4036 | 5y | “Have any of the these occurred in the past year (since your study child was 4). Some of these may be distressing to recall, but we hope you will let us know just how they affected you.” …”Your partner was emotionally cruel to you” | Psychological | Exposure |  |
| l4022 | 6y | “Have any of the these occurred since your study child’s 5^th^ birthday?” …”Your partner was physically cruel to you” | Physical | Exposure |  |
| l4036 | 6y | “Have any of the these occurred since your study child’s 5^th^ birthday?” …”Your partner was emotionally cruel to you” | Psychological | Exposure |  |
| n3035, n3037, n3039, n3041, n3043, n3045, n3045, n3049, n3051, n3053, n3055, n3057, n3059 | 8y* | 13 different examples of victimisation: e.g. “Has your husband/partner insulted or shamed you in front of others?” Other 12 relate to ‘pushed, grabbed, or shoved you’, ‘ever slapped you’, ‘ever shaken you’, ‘thrown at object at you that could hurt you’, ‘kicked, bitted, or hit you with a fist’, ‘ever hit or tried to hit you with something’, ‘ever physically twisted your arm’, ‘ever thrown or tried to throw you bodily’, ‘ever beaten you up (multiple blows)’, ‘ever tried to choke or strangle you’, ‘ever threatened you with a knife of other weapon’, ‘ever used a knife or other weapon on you’ | Psychological (first example), Physical (final twelve) | Exposure | 1 = Y, sometimes; Y, often  0 = N |
| p2022 | 9y | “Listed below are a number of events which may have brought changes in your life. Have any of these occurred since your study child’s 6^th^ birthday?” …”Your husband/partner was physically cruel to you” | Physical | Exposure | 1 = Y, when the study child was 6 or 7; Y, since the child’s 8^th^ birthday;  0 = N, did not happen in past 3 years |
| p2036 | 9y | “Listed below are a number of events which may have brought changes in your life. Have any of these occurred since your study child’s 6^th^ birthday?” …”Your husband/partner was emotionally cruel to you” | Psychological | Exposure |  |
| r5022 | 11y | “Listed below are a number of events which may have brought changes in your life. Have any of these occurred since your study child’s 9^th^ birthday?” …”Your husband/partner was physically cruel to you” | Physical | Exposure | 1 = Y, when the study child was 9 or 10; Y, since the child’s 11^th^ birthday;  0 = N, did not happen in this period |
| r5036 | 11y | “Listed below are a number of events which may have brought changes in your life. Have any of these occurred since your study child’s 9^th^ birthday?” …”Your husband/partner was emotionally cruel to you” | Psychological | Exposure |  |
| s3154 | 12y* | “In the past 3 months, have any of these happened?” …”hitting or slapping” | Physical | Exposure | 1 = Y, I did this; Y, he did this; Y, we both did this;  0 = N not at all |
| s3155 | 12y* | In the past 3 months, have any of these happened? …”thrown or broken things” | Physical | Exposure |  |
| s3202 s3211 s3216 s3219 | 12y* | “Below are attitudes and behaviours which people reveal in their close relationships. Please rate your husband/partner’s attitudes and behaviour towards you in recent times and tick the most appropriate box for each item. My husband/partner:” …”Wants to know exactly what I’m doing and where I am”, “Insists I do exactly as I’m told”, “Seeks to dominate me”, “Tends to control everything I do” | Controlling behaviours | Exposure in a supplementary analyses | 1 = Very true; Moderately true; Somewhat true;  0 = Not at all true |
| t3321 | 18y | “Listed below are a number of events which may have brought changes in your life. Have any of these occurred in the last year?” …”Your partner was physically cruel to you” | Physical | Exposure | 1 = Y affected a lot; Y moderately affected; Y mildly affected; Y but did not affect me;  0 = N did not happen at all |
| t3335 | 18y | “Listed below are a number of events which may have brought changes in your life. Have any of these occurred in the last year?” …”Your partner was emotionally cruel to you” | Psychological | Exposure |  |

ALSPAC = Avon Longitudinal Study of Parents & Children

*Measures used in sensitivity analyses when exposure restricted to acts-based measures.

**Box S2: Intimate Partner Violence and Abuse (IPVA) section of questionnaire delivered to ALSPAC ‘children’ (as young adults) at age 21**


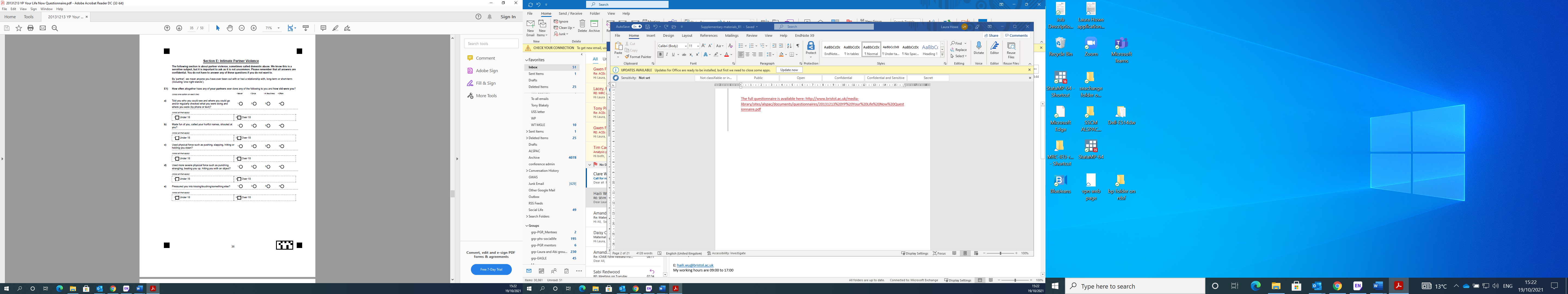


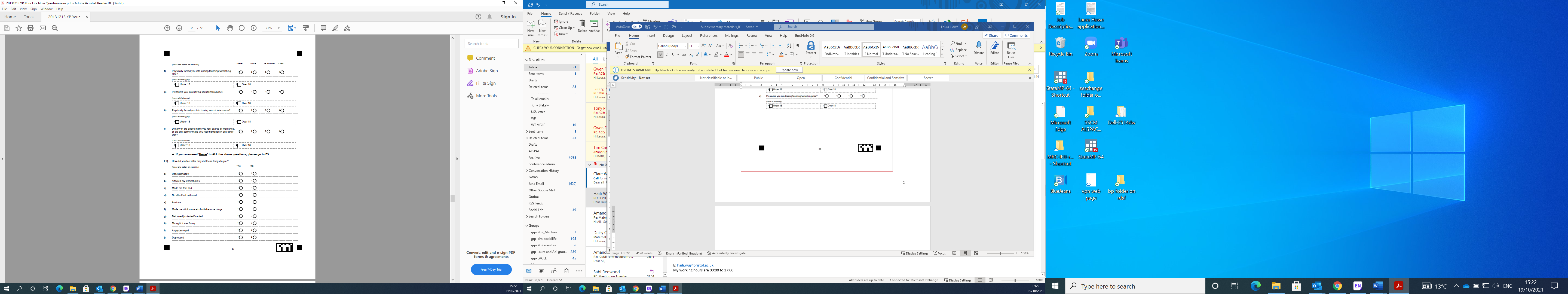


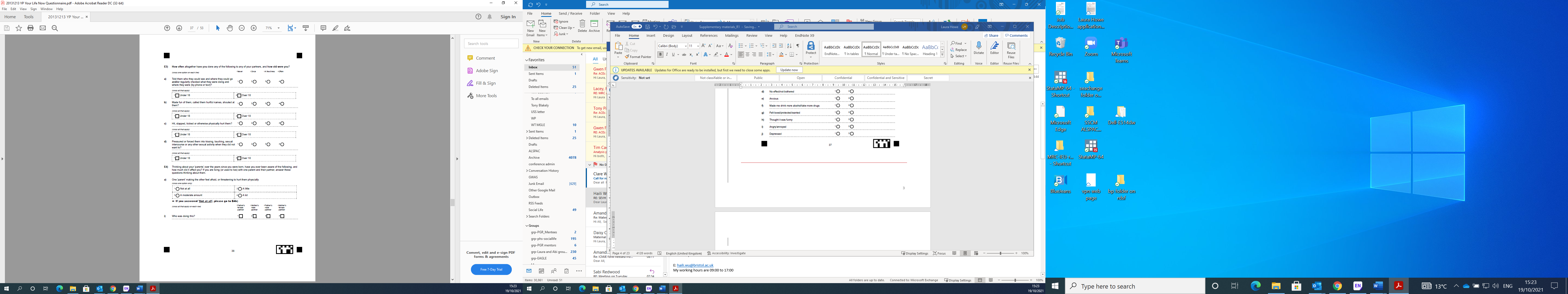


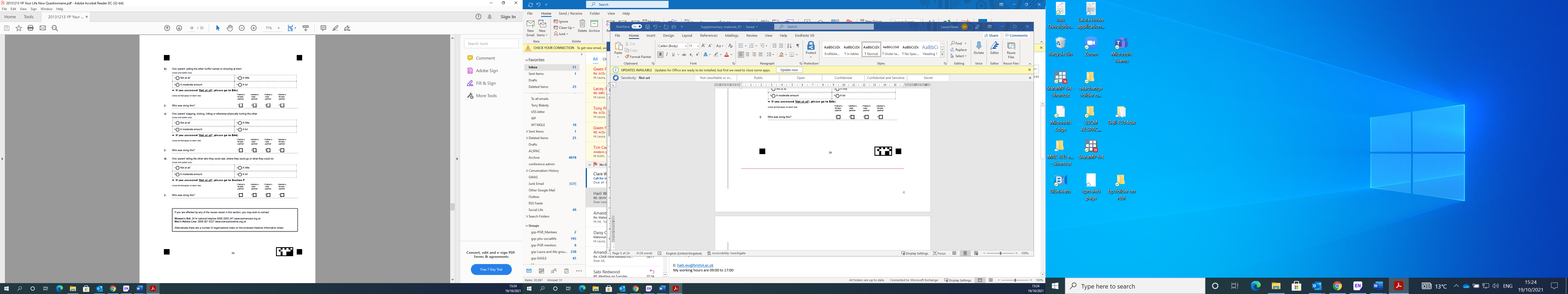


**Table S2: Variables used to capture covariates considered in analysis and imputation models**

| **Covariate** | **ALSPAC variable(s)** | **Age of ALSPAC child** | **Values, and how analysed** |
| --- | --- | --- | --- |
| Marital status | a525 | 8 weeks gestation | Binary variable:  0 = Never married; Widowed; Divorced; Separated  1 = 1st marriage; Marriage 2 or 3 |
| Parental IPVA in pregnancy | b592, b607, pb182a, pb196a | 18 weeks gestation | Binary variable:  0 = N did not happen at all, for all four variables  1 = Y affected a lot; Y moderately affected; Y mildly affected; Y but did not affect me; for any of the four variables  Coded as missing if at least two of the four variables had missing values. |
| Parity | b032 | 18 weeks gestation | Continuous numeric variable, any number >3 replaced with ‘3’ due to sparse high numbers. |
| Mother’s Edinburgh ‘post-natal’ depression score | c600 | 32 weeks gestation | Numerical discrete variable (score from 0-29) |
| Highest household education | c645, pb325 | 32 weeks (c645) and 18 weeks (pb325) gestation | Binary variable, taking highest value of c645 (mother) and pb325 (partner):  0 = <O-level; O-level  1 = A-level; Degree or above |
| Household social class | c755, c765 | 32 weeks gestation | Binary variable, taking highest value of c755 (mother) and c765 (partner’s social class, mother-reported), based on employment status:  0 = I; II; III; IV non-managerial  1 = IV managerial; V |
| Ethnicity | c800 | 32 weeks gestation | Binary variable:  0 = White;  1 = Person of Colour  Eventually wasn’t included due to small numbers for ‘Person of Colour’, models wouldn’t converge. |
| Maternal age | mz028b | Birth | Continuous numeric variable (years) |
| Maternal smoking in pregnancy | b663 | 18 weeks gestation | Binary variable:  1 = Yes, cigarettes, cigars, pipe, or other  0 = No |
| Birthweight of ALSPAC child | kz030 | Birth | Continuous numeric variable (kilograms) |
| Adverse Childhood Experiences (ACEs): Emotional abuse; emotional neglect; physical abuse; sexual abuse; bullying; parental mental health problems, parental substance abuse, parental criminal conviction, parental separation | Listed in Supplemental Table 1 of Houtepen *et al*.(6) | 0-16 years | Binary variable for each ACE construct  0 = ACE not present  1 = ACE present |

ALSPAC = Avon Longitudinal Study of Parents and Children; IPVA = Intimate Partner Violence and Abuse

**Box S3: Details on deriving Population Attributable Fractions (PAFs), Risk Differences (RDs) and their Confidence Intervals (CIs)**

*For single exposures (e.g. of any Parental Psychological IPVA):*

PAFs were calculated as:

$$PAF=p(1-\frac{1}{RR})\times100$$

Where $p$ represents the sample prevalence of the exposure of interest (e.g. parental psychological IPVA) among those with the outcome (e.g. young adults exposed to IPVA victimisation), and $RR$ is the relative risk of the outcome between those with and without that exposure of interest, estimated from adjusted Model A (as PAFs are assumed to be derived from RRs representing causal relationships). 95% confidence intervals were calculated as per the equation above, where $RR$ was replaced with the lower and upper limits of the 95% confidence interval for the $RR$ (the ‘inverse method’).(7)

RDs were calculated as:

$$RD=(P_{1}-P_{0})\times100$$

Where $P_{1}$ and $P_{0}$ are the probabilities of the outcome (i.e. young adults exposed to IPVA victimisation) among those with and without the exposure of interest (any Parental IPVA), as estimated by adjusted Model A. 95% confidence intervals were calculated where the standard error of $RD$ was calculated as $\sqrt{Var\left( P_{1} \right)+Var(P_{0})}$.

*For combined exposures (e.g. of Parental Psychological IPVA + Maltreatment):*

‘Category-specific’ PAFs (as per Rockhill *et al*, 1998), were calculated:

$${PAF}_{i}=p_{i}(1-\frac{1}{{RR}_{i}})\times100$$

Where $i = 0,1, 2$; 0 represents no Parental IPVA (e.g. no Parental Psychological IPVA, whether Maltreatment present or not); 1 Parental IPVA but not the other exposure of interest (e.g. Parental Psychological IPVA not Maltreatment); 2 both Parental IPVA and other exposure of interest (e.g. Parental Psychological IPVA + Maltreatment).

In this example, ${PAF}_{2}$ would be the PAF estimate for Parental Psychological IPVA + Maltreatment, $p_{2}$ the combined prevalence of Parental Psychological IPVA and Maltreatment, among those with the outcome (e.g. young adults exposed to IPVA victimisation). ${RR}_{2}$ would be the relative risk of the outcome for those with Parental Psychological IPVA + Maltreatment vs. no Parental Psychological IPVA, whether Maltreatment present or not, from a model adjusted for the same covariates as in Adjusted Model A (i.e. adjustments that, unliked Adjusted Model B, still allow a causal interpretation of the RR).

The three-level categorical variable that the RR represents is derived in the above way to enforce a common reference category between the RRs used to derive the PAF for Any Parental IPVA, and PAFs for pairwise combinations of Parental IPVA and other factors of interest, respectively. This is so they may be validly compared. That is, the reference category in all cases is no Parental IPVA.

RDs were calculated as:

$${RD}_{2}=(P_{2}-P_{0})\times100$$

Where $P_{2}$ and $P_{0}$ are the probabilities of the outcome (i.e. young adults exposed to IPVA victimisation) among those with both exposures of interest, and no Parental IPVA, respectively, from the same model as used to estimate ${RR}_{2}$. 95% confidence intervals were calculated where the standard error of $RD$ was calculated as $\sqrt{Var\left( P_{2} \right)+Var(P_{0})}$.

**Table S3: Proportions of missing data in exposures, outcomes, and covariates, prior to multiple imputation**

| **Parents (age of child/young adult when measured)** | **Men (n=2,104)** | **Women (n=1,139)** |
| --- | --- | --- |
| Mother’s marital status in pregnancy | 5.7 | 6.7 |
| Sexual orientation (by age 7) | 82.7 | 70.9 |
| Smoked in pregnancy | 5.0 | 6.6 |
| Age of mother at delivery in years (birth) | 4.5 | 5.0 |
| Parity (birth) | 5.7 | 7.7 |
| Household highest education level (birth) | 6.5 | 8.6 |
| Household highest social class (birth) | 10.1 | 11.9 |
| Any IPVA (0-18y) |  | 18.9 |
| Physical | 13.4 | 18.9 |
| Psychological | 13.4 | 18.9 |
| Controlling behaviours | 13.4 | 18.9 |
| **Child/young adult (age when measured)** |  |  |
| Ethnicity (birth) | 1.0 | 1.0 |
| Sexual orientation (21y)* | 23.2 | 26.6 |
| Weight (g, birth) | 5.9 | 6.3 |
| Adverse Childhood Experiences (ACEs; 0-16y) |  |  |
| Maltreatment | 21.9 | 27.6 |
| Emotional abuse | 14.8 | 20.7 |
| Emotional neglect | 15.0 | 21.4 |
| Physical abuse | 15.4 | 20.3 |
| Sexual abuse | 8.1 | 11.8 |
| Bullying | 12.9 | 17.5 |
| Parental mental health problems | 13.5 | 20.0 |
| Parental substance misuse | 15.4 | 22.2 |
| Parental criminal conviction | 14.1 | 20.4 |
| Parental divorce/separation | 19.0 | 25.2 |

N.B. Since one study inclusion criterion was that the individual responded to the IPVA questionnaire at age 21, none of the IPVA outcomes are missing.

**Table S4: Association of maternal IPVA victimisation with young adult IPVA*, overall (any maternal IPVA) and by IPVA type (e.g. psychological), in complete case (n=953 to 1878) vs. imputed data (n, women=2104, men=1139; including when exposure restricted**)**

|  |  | **Young adult IPVA victimisation** | | | |
| --- | --- | --- | --- | --- | --- |
| **Parental IPVA** |  | **Women**  **RR (95% CI)** | | **Men**  **RR (95% CI)** | |
| **Any (age 2-18)** | Imputed | 1.13 | (0.98 to 1.30) | 1.00 | (0.79 to 1.25) |
|  | Complete case (women=1878; men=1052) | 1.13 | (0.98 to 1.30) | 1.04 | (0.83 to 1.30) |
|  | Imputed (restricted exposure) | 0.99 | (0.85 to 1.16) | 0.93 | (0.72 to 1.20) |
| **Physical (age 2-18)** | Imputed | 0.94 | (0.80 to 1.10) | 1.10 | (0.85 to 1.41) |
|  | Complete case (women=1657;men=953) | 0.92 | (0.77 to 1.10) | 1.13 | (0.87 to 1.48) |
|  | Imputed (restricted exposure) | 0.89 | (0.73 to 1.07) | 1.02 | (0.76 to 1.37) |
| **Psych (any; age 2-18)** | Imputed | 1.17 | (1.02 to 1.35) | 1.06 | (0.84 to 1.34) |
|  | Complete case (women=1657; men=953) | 1.20 | (1.03 to 1.39) | 1.12 | (0.87 to 1.43) |
|  | Imputed (restricted exposure) | 1.04 | (0.88 to 1.22) | 1.00 | (0.75 to 1.32) |
| **Coercive controlling behaviours (age 12)^** | Imputed | 0.94 | (0.73 to 1.22) | 1.19 | (0.81 to 1.74) |
|  | Complete case (women=1657; men=953) | 0.98 | (0.74 to 1.29) | 1.31 | (0.88 to 1.98) |
|  |  | **Young adult IPVA perpetration** | | | |
|  |  | **Women** | | **Men** | |
| **Any (age 2-18)** | Imputed | 1.04 | (0.86 to 1.26) | 1.22 | (0.91 to 1.65) |
|  | Complete case (women=1878; men=1052) | 0.99 | (0.82 to 1.19) | 1.24 | (0.92 to 1.65) |
|  | Imputed (restricted exposure) | 0.91 | (0.75 to 1.12) | 1.22 | (0.88 to 1.69) |
| **Physical (age 2-18)** | Imputed | 0.99 | (0.79 to 1.23) | 1.45 | (1.05 to 2.00) |
|  | Complete case (women=1657;men=953) | 0.91 | (0.72 to 1.15) | 1.55 | (1.12 to 2.15) |
|  | Imputed (restricted exposure) | 0.88 | (0.69 to 1.13) | 1.57 | (1.11 to 2.22) |
| **Psych (any; age 2-18)** | Imputed | 1.08 | (0.89 to 1.3) | 1.18 | (0.86 to 1.62) |
|  | Complete case (women=1657; men=953) | 1.06 | (0.87 to 1.30) | 1.16 | (0.84 to 1.60) |
|  | Imputed (restricted exposure) | 0.88 | (0.70 to 1.11) | 1.04 | (0.72 to 1.51) |
| **Coercive controlling behaviours (age 12)^** | Imputed | 0.88 | (0.6 to 1.27) | 1.28 | (0.76 to 2.14) |
|  | Complete case (women=1657; men=953) | 0.94 | (0.65 to 1.37) | 1.43 | (0.85 to 2.40) |

*Adjusted for age of mother at delivery (in years), parity (analysed as 1, 2, 3+), birthweight (in grams), whether mother smoked in pregnancy or not, mother’s highest education (>=A-level vs. < A-level), household social class (I; II; III non-managerial; III managerial; IV or V), and any parental IPVA during pregnancy (yes/no).

**Only including acts-based measures of maternal IPVA victimisation, at ages 8 and 12 (further detail provided in Table S1).

^For controlling behaviours, estimates for ‘imputed (restricted exposure)’ Identical to imputed data row, as originally only captured through acts-based measures.

CI = Confidence Interval; RR = Relative Risk

**Table S5: Associations of parental IPVA with young adult IPVA victimisation, overall (any parental IPVA) and by IPVA type (e.g. psychological)**

| **Women** | **Parental IPVA type (when measured)** | **Level** | **% with outcome** | **Crude RR** | **(95% CI)** | **Adjusted Model A RR** | **(95% CI)** | **Adjusted Model B RR** | **(95% CI)** |
| --- | --- | --- | --- | --- | --- | --- | --- | --- | --- |
|  | **Any (age 2-18)** | No | 29.6 |  |  |  |  |  |  |
|  |  | Yes | 34.9 | 1.18 | (1.03 to 1.35) | 1.13 | (0.98 to 1.30) | 1.05 | (0.90 to 1.22) |
|  | **Physical (age 2-18)** | No | 32 |  |  |  |  |  |  |
|  |  | Yes | 32.3 | 1.01 | (0.86 to 1.18) | 0.94 | (0.80 to 1.10) | 0.87 | (0.74 to 1.03) |
|  | **Psych (any; age 2-18)** | No | 29.4 |  |  |  |  |  |  |
|  |  | Yes | 36.1 | 1.23 | (1.07 to 1.41) | 1.17 | (1.02 to 1.35) | 1.09 | (0.94 to 1.26) |
|  | **Controlling behaviours (age 12)** | No | 32.2 |  |  |  |  |  |  |
|  |  | Yes | 31 | 0.96 | (0.74 to 1.25) | 0.94 | (0.73 to 1.22) | 0.89 | (0.68 to 1.15) |
|  |  |  |  |  |  |  |  |  |  |
| **Men** | **Any (age 2-18)** | No | 23.4 |  |  |  |  |  |  |
|  |  | Yes | 24.2 | 1.03 | (0.83 to 1.29) | 1.00 | (0.79 to 1.25) | 0.92 | (0.72 to 1.17) |
|  | **Physical (age 2-18)** | No | 22.9 |  |  |  |  |  |  |
|  |  | Yes | 26.6 | 1.16 | (0.90 to 1.50) | 1.10 | (0.85 to 1.41) | 1.00 | (0.76 to 1.31) |
|  | **Psych (any; age 2-18)** | No | 22.9 |  |  |  |  |  |  |
|  |  | Yes | 25.3 | 1.10 | (0.87 to 1.39) | 1.06 | (0.84 to 1.34) | 0.99 | (0.77 to 1.28) |
|  | **Controlling behaviours (age 12)** | No | 23.4 |  |  |  |  |  |  |
|  |  | Yes | 29.3 | 1.25 | (0.85 to 1.84) | 1.19 | (0.81 to 1.74) | 1.15 | (0.77 to 1.70) |

CI = Confidence Interval; Psych = Psychological; RR = Relative Risk

Each relative risk and confidence interval estimated from a separate modified Poisson model (i.e. the table represents estimates from 24 different models).

Model A adjusted for age of mother at delivery (in years), parity (analysed as 1, 2, 3+), birthweight (in grams), whether mother smoked in pregnancy or not, mother’s highest education (>=A-level vs. < A-level), household social class (I; II; III non-managerial; III managerial; IV or V), and any parental IPVA during pregnancy (yes/no).

Model B adjusted for same covariates as in Model A + 9 binary indicators of other Adverse Childhood Experiences (parental substance abuse, parental mental health problems, parental criminal conviction, parental separation, child emotional neglect, child emotional abuse, child physical abuse, child sexual abuse, child bullying).

**Table S6: Associations of parental IPVA with young adult IPVA perpetration, overall (any parental IPVA) and by IPVA type (e.g. psychological)**

| **Women** | **Parental IPVA type** | **Level** | **% with outcome** | **Crude RR** | **(95% CI)** | **Adjusted Model A RR** | **(95% CI)** | **Adjusted Model B RR** | **(95% CI)** |
| --- | --- | --- | --- | --- | --- | --- | --- | --- | --- |
|  | **Any (age 2-18)** | No | 19.7 |  |  |  |  |  |  |
|  |  | Yes | 22 | 1.12 | (0.93 to 1.34) | 1.04 | (0.86 to 1.26) | 0.96 | (0.78 to 1.18) |
|  | **Physical (age 2-18)** | No | 20.3 |  |  |  |  |  |  |
|  |  | Yes | 22.2 | 1.09 | (0.88 to 1.35) | 0.99 | (0.79 to 1.23) | 0.92 | (0.73 to 1.15) |
|  | **Psych (any; age 2-18)** | No | 19.6 |  |  |  |  |  |  |
|  |  | Yes | 22.6 | 1.15 | (0.96 to 1.38) | 1.08 | (0.89 to 1.3) | 0.98 | (0.80 to 1.20) |
|  | **Controlling behaviours (age 12)** | No | 20.9 |  |  |  |  |  |  |
|  |  | Yes | 19.2 | 0.91 | (0.63 to 1.32) | 0.88 | (0.6 to 1.27) | 0.81 | (0.56 to 1.18) |
|  |  |  |  |  |  |  |  |  |  |
| **Men** | **Any (age 2-18)** | No | 13.8 |  |  |  |  |  |  |
|  |  | Yes | 17.8 | 1.29 | (0.96 to 1.73) | 1.22 | (0.91 to 1.65) | 1.15 | (0.84 to 1.57) |
|  | **Physical (age 2-18)** | No | 13.8 |  |  |  |  |  |  |
|  |  | Yes | 21.3 | 1.54 | (1.13 to 2.11) | 1.45 | (1.05 to 2.00) | 1.36 | (0.97 to 1.89) |
|  | **Psych (any; age 2-18)** | No | 14.3 |  |  |  |  |  |  |
|  |  | Yes | 17.7 | 1.24 | (0.91 to 1.69) | 1.18 | (0.86 to 1.62) | 1.13 | (0.81 to 1.58) |
|  | **Controlling behaviours (age 12)** | No | 15.3 |  |  |  |  |  |  |
|  |  | Yes | 19.7 | 1.28 | (0.77 to 2.12) | 1.28 | (0.76 to 2.14) | 1.22 | (0.71 to 2.09) |

CI = Confidence Interval; Psych = Psychological; RR = Relative Risks

Each relative risk and CI estimated from a separate log-linear model (i.e. the table represents estimates from 24 different models).

Model A adjusted for age of mother at delivery (in years), parity (analysed as 1, 2, 3+), birthweight (in grams), whether mother smoked in pregnancy or not, mother’s highest education (>=A-level vs. < A-level), household social class (I; II; III non-managerial; III managerial; IV or V), and any parental IPVA during pregnancy (yes/no).

Model B adjusted for same covariates as in Model A + 9 binary indicators of other Adverse Childhood Experiences (parental substance abuse, parental mental health problems, parental criminal conviction, parental separation, child emotional neglect, child emotional abuse, child physical abuse, child sexual abuse, child bullying).

**References**

1. Harris PA, Taylor R, Thielke R, Payne J, Gonzalez N, Conde JG. Research electronic data capture (REDCap)--a metadata-driven methodology and workflow process for providing translational research informatics support. J Biomed Inform. 2009;42(2):377-81.

2. Field CA, Caetano R. Ethnic differences in intimate partner violence in the U.S. general population: the role of alcohol use and socioeconomic status. Trauma Violence Abuse. 2004;5(4):303-17.

3. Cho H. Racial differences in the prevalence of intimate partner violence against women and associated factors. J Interpers Violence. 2012;27(2):344-63.

4. Capaldi DM, Knoble NB, Shortt JW, Kim HK. A Systematic Review of Risk Factors for Intimate Partner Violence. Partner Abuse. 2012;3(2):231-80.

5. Yakubovich AR, Stöckl H, Murray J, Melendez-Torres GJ, Steinert JI, Glavin CEY, et al. Risk and protective factors for intimate partner violence against women: Systematic review and meta-analyses of prospective–longitudinal studies. American Journal of Public Health. 2018;108(7):e1-e11.

6. Houtepen LC, Heron J, Suderman MJ, Tilling K, Howe LD. Adverse childhood experiences in the children of the Avon Longitudinal Study of Parents and Children (ALSPAC). Wellcome Open Research. 2018;3(0):106-.

7. Camacho-García-Formentí; D, Zepeda-Tello R. Introduction to the pifpaf package: Estimating Potential Impact and Population Attributable Fractions from Cross-Sectional data 2019 [Available from: <http://cran.nexr.com/web/packages/pifpaf/vignettes/Introduction_to_pifpaf_package.html#deciding-on-the-confidence-interval>.
